# Effect of an Ethanol Extract of *Scutellaria baicalensis* on Relaxation in Corpus Cavernosum Smooth Muscle

**DOI:** 10.1155/2012/148929

**Published:** 2011-12-22

**Authors:** Xiang Li, Hyun Cheol Oh, Su Bin Son, Yun Jung Lee, Dae Gill Kang, Ho Sub Lee

**Affiliations:** ^1^Professional Graduate School of Oriental Medicine and College of Oriental Medicine, Wonkwang University, Iksan, Jeonbuk 570-749, Republic of Korea; ^2^Hanbang Body-fluid Research Center, Wonkwang University, Iksan, Jeonbuk 570-749, Republic of Korea; ^3^College of Medical and Life Sciences, Silla University, San 1-1 Gwaebeop-dong, Sasang-gu, Busan 617-736, Republic of Korea

## Abstract

*Aims of study*. The aim of the present study was to investigate whether an ethanol extract of *Scutellaria baicalensis* (ESB) relaxes penile corpus cavernosum muscle in organ bath experiments. *Materials and methods*. Changes in tension of cavernous smooth muscle strips were determined by penile strip chamber model and in penile perfusion model. Isolated endothelium-intact rabbit corpus cavernosum was precontracted with phenylephrine (PE) and then treated with ESB. *Results*. ESB relaxed penile smooth muscle in a dose-dependent manner, and this was inhibited by pre-treatment with N^G^-nitro-l-arginine methyl ester (l-NAME), a nitric oxide (NO) synthase inhibitor, and 1H-[1, 2, 4]-oxadiazolo-[4,3-*α*]-quinoxalin-1-one (ODQ), a soluble guanylyl cyclase (sGC) inhibitor. ESB-induced relaxation was significantly attenuated by pretreatment with tetraethylammonium (TEA), a nonselective K^+^ channel blocker, and charybdotoxin, a selective Ca^2+^-dependent K^+^ channel inhibitor. ESB increased the cGMP levels of rabbit corpus cavernosum in a concentration-dependent manner without changes in cAMP levels. In a perfusion model of penile tissue, ESB also relaxed penile corpus cavernosum smooth muscle in a dose-dependent manner. *Conclusion*. Taken together, these results suggest that ESB relaxed rabbit cavernous smooth muscle via the NO/cGMP system and Ca^2+^-sensitive K^+^ channels in the corpus cavernosum.

## 1. Introduction

Penile erection is a hemodynamic process induced by both increased arterial inflow and restricted venous outflow and is coordinated with smooth muscle relaxation within the corpus cavernosum under neural control [1, 2]. Penile tumescence and detumescence are regulated by a complex neurophysiological process involving the relaxation and contraction, respectively, of the corpus cavernosum. Erection occurs when the penile arteries and corpus cavernosal smooth muscle are relaxed by nitric oxide (NO) released from nerve endings and endothelial cells [3, 4]. The activation of the soluble guanylyl cyclase (sGC) by NO results in an increase of intracellular cGMP production which stimulates the cGMP-dependent protein kinase (PKG) that, in turn, lowers intracellular Ca^2+^ concentrations and/or the Ca^2+^ sensitivity of the contractile machinery to produce relaxation [5]. In penile tissue, cGMP is predominantly metabolized by phosphodiesterase type 5 (PDE 5). This enzyme is the most prominent target of the NO/cGMP signalling cascade identified to date for pharmacological intervention in erectile dysfunction. Therefore, the NO/cGMP pathway plays a key role in penile erection.

Penile erection is a result of increased intracavernous blood flow and pressure (ICP). Thus, pharmacologically active agents that are capable of increasing the blood flow and pressure can also ameliorate erectile dysfunction (ED). Another candidate for the treatment of ED is the K^+^ channel activator. The large conductance Ca^2+^-sensitive K^+^ (BK_Ca_) channels, ATP-sensitive potassium (K_ATP_) channels, and voltage-activated K^+^ (K_V_) channels are important modulators of cavernous smooth muscle tone.

Recently, in the course of searching for potential therapeutic agents from traditional oriental medicines for the treatment of ED, we found that extracts of *Scutellaria baicalensis* Georgi (Hwang Geum in Korea; Huang Qin in China) elicit cavernous smooth muscle relaxation. The dried root of *S. baicalensis* (Solanaceae) is an important traditional medicine widely used in China, Korea, and Japan because of its therapeutic effectiveness in the treatment of dysentery, pyrexia, jaundice, and carbuncles [6, 7]. It is known to have a multitude of pharmacological effects, including anti-inflammatory [8], antidiabetic [9], antiviral [10], antihypertension [6], antioxidant [8, 11, 12], and anticancer [13] effects. The roots are rich in baicalein, baicalin, wogonin, chrysin, and wogonoside, which exert anti-inflammatory and antioxidant activities. Baicalein induces relaxation in rat mesenteric artery [14]. Moreover, baicalein and wogonin inhibit collagen deposition [15]. In addition, studies have reported that long-term baicalein administration ameliorates metabolic disorder [16]. However, to the best of our knowledge, the effect of *S. baicalensis* on ED has not yet been defined. Therefore, the purpose of the present study was to investigate the cavernous smooth muscle relaxant activity of an ethanol extract of the root of *S. baicalensis* (ESB) and its mechanism of action. Moreover, this study was designed to investigate the effect of ESB on erectile function and to explore the association of NO/cGMP pathway in rabbit corpus cavernosum.

## 2. Materials and Methods

### 2.1. Extraction of *S. baicalensis*

The root of *S. baicalensis* (Solanaceae) was purchased from the Herbal Medicine Co-operative Association Iksan, Korea in July 2008. Herbarium voucher specimens of ESB (DH-127) were prepared and deposited in the Herbarium of the Professional Graduate School of Oriental Medicine, Wonkwang University, Iksan, Korea. The dried root of *S. baicalensis* (600 g) was extracted in 3 L of ethanol (99%) at 24°C for 7 days, filtered through Whatman No. 2 filter paper, and concentrated using a rotary evaporator. After lyophilized using freeze-drier, the yield of the ethanol extract (ESB) was 2.02% of the plant powder and dissolved in dimethyl sulfoxide (DMSO). The final concentration of DMSO was less than 0.1%. 

### 2.2. High-Performance Liquid Chromatography Fingerprinting and Nuclear Magnetic Resonance Analysis

A portion of the extract (2.3 g) was subjected to octadecyl functionalized silica gel flash column (5 × 40 cm; particle size, 75 *μ*m) chromatography. The column was eluted with a stepwise gradient of methanol (MeOH) in H_2_O (from 20% to 100% with 20% increments; 5 fractions of 500 mL each), followed by 500 mL of 50% MeOH in CH_2_Cl_2_, resulting in the collection of 6 fractions (Fr. 1–6). A portion of the fraction that was eluted with 40% MeOH in H_2_O was further purified by semipreparative reversed-phase high-performance liquid chromatography (HPLC; Agilent prep-C_18_ column [21.2 × 150 mm; particle size, 5 *μ*m]; 5 mL/min; detection wavelength, 254 nm) by eluting with a gradient of 30–58% MeOH in H_2_O (0.1% formic acid) over 16 min to yield **1 **(*t*_*R*_ = 18.3 min). A portion of the fraction that eluted with 80% MeOH in H_2_O was further purified by semipreparative reversed-phase HPLC using an identical column, but eluting with a gradient of 60–73% MeOH in H_2_O (0.1% formic acid) over 25 min to yield **2 **(*t*_*R*_ = 19.4 min), **3 **(*t*_*R*_ = 20.5 min), and **4 **(*t*_*R*_ = 25.0 min). Nuclear magnetic resonance (NMR) spectra (1D and 2D) were recorded using a JEOL JNM ECP-400 spectrometer (400 MHz for ^1^H and 100 MHz for ^13^C), and chemical shifts were referenced relative to the corresponding residual solvent signals. Heteronuclear single-quantum coherence (HSQC) and heteronuclear multiple bond coherence (HMBC) experiments were optimized for ^1^ *J*_CH_ = 140 Hz and ^*n*^ *J*_CH_ = 8 Hz, respectively. The chromatographic fingerprint of the ethanol extract of *S. baicalensis *was performed on a YOUNGLIN system (YOUNGLIN Instrument, Korea) equipped with a YOUNGLIN UV detector (UV 730D) and Zam3000 Evaporative Light Scattering Detector (Schambeck SFD GmbH, Bad Honnef, Germany). Chromatographic separation was carried out on an Eclipse XDB-C18 column (4.6 mm × 150 mm; particle size, 5 *μ*m) at room temperature with an injection volume of 50 *μ*L using a gradient elution of 10% MeOH in water (0.1% formic acid) to 100% MeOH over 60 min. Peaks were detected simultaneously at both 210 and 254 nm by UV detection.

### 2.3. Organ Bath Experiments

 The animal procedures were in strict accordance with the National Institute of Health Guide for the Care and Use of Laboratory Animals and were approved by the Institutional Animal Care and Utilization committee for Medical Science of Wonkwang University. Adult (4-5-month-old) male New Zealand White rabbits (3.0–3.5 kg) were sacrificed by an overdose of pentobarbitone injected into the marginal vein of the ear. The corpus cavernosum strips were mounted in tissue baths containing 5 mL Krebs solution (118 mM NaCl, 4.7 mM KCl, 1.1 mM MgSO_4_, 1.2 mM KH_2_PO_4_, 1.5 mM CaCl_2_, 25 mM NaHCO_3_, and 10 mM glucose) at 37°C equilibrated in 95% O_2_ and 5% CO_2_. The strips were suspended with silk ties to a force-displacement transducer (Grass FT 03; Grass Instrument Co., Quincy, Mass, USA), and isometric tension was recorded on the physiograph. After a 60-min equilibration, the strips of corpus cavernosum were loaded with a resting tension of 1 g and then contracted with 1 *μ*M phenylephrine (PE) to obtain stable peak response. Once stable peak response to PE had been obtained, the cavernosal strips were exposed to cumulative doses of the testing agent, and the responses were recorded. To define the mechanisms by which ESB relaxes cavernosal strips, the strips were exposed to various modulating agents (indomethacin, diltiazem, atropine, propranolol, etc.) for 20 min, and then cavernosal smooth muscle relaxation was carried out by cumulative addition of ESB. The effect of vehicle, <0.1% dimethylsulfoxide (DMSO), was also tested. After each test, the cavernosal strips were washed 3 times with fresh Krebs solution and allowed to equilibrate for 30 min.

### 2.4. Measurement of cGMP and cAMP Levels in Corpus Cavernosum

To determine the effect of ESB on cGMP and cAMP levels, we used healthy male rabbit corpus cavernosum. After equilibration of the cavernosal strips for 60 min in 2 mL of Krebs solution gassed with 95% O_2_/5% CO_2_ at a constant temperature of 37°C in a shaking water bath, the strips were incubated for an additional 20 min in the presence of 100 *μ*M 3-isobutyl-1-methylxanthine (IBMX) before the addition of 1 *μ*M PE. After the cavernosal strips were subjected to ESB in the presence or absence of modulators for 5 min, the reactions were stopped by freezing the tissues in liquid N_2_. The tissues were homogenized with a polytron homogenizer in 500 *μ*L of 6% trichloroacetic acid (TCA) solution. The homogenates were centrifuged at 13,000 rpm for 15 min, and the supernatants were collected. The protein concentration was determined by the method of Bradford (Bradford, 1976). The supernatant was extracted 3 times with water-saturated diethylether and concentrated using a Speed-Vac concentrator (Savant Instrument, Farmingdale, NY, USA). cGMP and cAMP content was measured by an equilibrated radioimmunoassay by using a method as previously described [17]. In brief, standards or samples were introduced in a final volume of 100 *μ*L of 50 mM sodium acetate buffer (pH 4.85). Then, 100 *μ*L each diluted cGMP or cAMP antiserum (Calbiochem-Novabiochem Co., San Diego, Calif, USA) and iodinated cGMP or cAMP (10,000 cpm/100 *μ*L) were added in succession and incubated for 24 h at 4°C, respectively. The bound form was separated from the free form by charcoal suspension. Results were expressed as picomoles of cGMP and cAMP per milligram of protein per minute.

### 2.5. Measurement of Isometric Tension in Perfused Penile Tissue Model

New Zealand White rabbits weighing (3.0–3.5 kg) were used. Rabbits were anesthetized by injecting ketamine-HCl (50 mg/kg). Whole penis was incised from hip bone with extreme care to protect the endothelium from inadvertent damage. Dissected penis carefully moved to oxygenated saline and separate penis muscle from urethra. The perfused penis model was as follows [18]. Two small cannulas were buried proximal crus of penis and fixed. The end of glans was cut to make the perfusate flow out. The cannulated penis was placed in a temperature-regulated organ chamber. The upper part of cannula fixed on force transducer (PowerLab/8sp, ADInstruments, Australia) to record tension. The penis was perfused with Krebs buffer solution by means of a peristaltic pump (0.25 mL/min). The composition of the buffer was as follows (in mM): 118 NaCl, 4.7 KCl, 2.5 CaCl_2_, 1.2 MgCl_2_, 25 NaHCO_3_, 10.0 glucose (adjust to pH 7.4), and 0.1% bovine serum albumin (BSA). The temperature of organ bath was maintained with circulator at 37°C. The perfusate was prewarmed to 37°C by passage through a water bath and equilibrated with oxygen by passage through silicone tubing in a gas mixing chamber. After stabilization for 2 hour, the penis was perfused for 60 min and collected at 2 min intervals for analyses.

### 2.6. Reagents

Sodium chloride, potassium chloride, calcium chloride, magnesium chloride, sodium bicarbonate, glucose, bovine serum albumin, sodium acetate, theophylline, sodium azide, potassium phosphate monobasic, potassium phosphate dibasic, phenyl mercuric acetate, charcoal, acetylcholine chloride (ACh), phenylephrine HCl (PE), N^G^-nitro-L-arginine methyl ester (l-NAME), 1H-[1, 2, 4]-oxadiazole-[4,3-*α*]-quinoxalin-1-one (ODQ), indomethacin, glibenclamide, tetraethylammonium chloride (TEA), 3-isobutyl-1-methyl-xanthine (IBMX), atropine, and (±)-propranolol HCl were purchased from Sigma Chemical Co (St. Louis, Mo, USA), and 4-aminopyridine (4-AP) was purchased from Biomol (Plymouth Meeting, Pa, USA). The following reference materials were obtained from the sources specified: anti-cGMP and anti-cAMP (Merck Bioscience Calbiochem, USA) and ^125^I-Na (Amersham Biosciences, Sweden). Control experiments demonstrated that the concentration of dimethyl sulfoxide (DMSO, 0.1%) had no significant effect on cavernous smooth muscle tone. 

### 2.7. Statistical Analyses

Relaxant responses are expressed as percentage relaxation of the PE precontraction levels unless otherwise described in the figure legends. Results were expressed as mean ± S.E. The statistical significance of difference between the group means was determined using one-way analysis of variance (ANOVA) and Student's *t*-test.

## 3. Results

### 3.1. Identification of Compounds in Extracts of S. baicalensis

**Figure 1:**
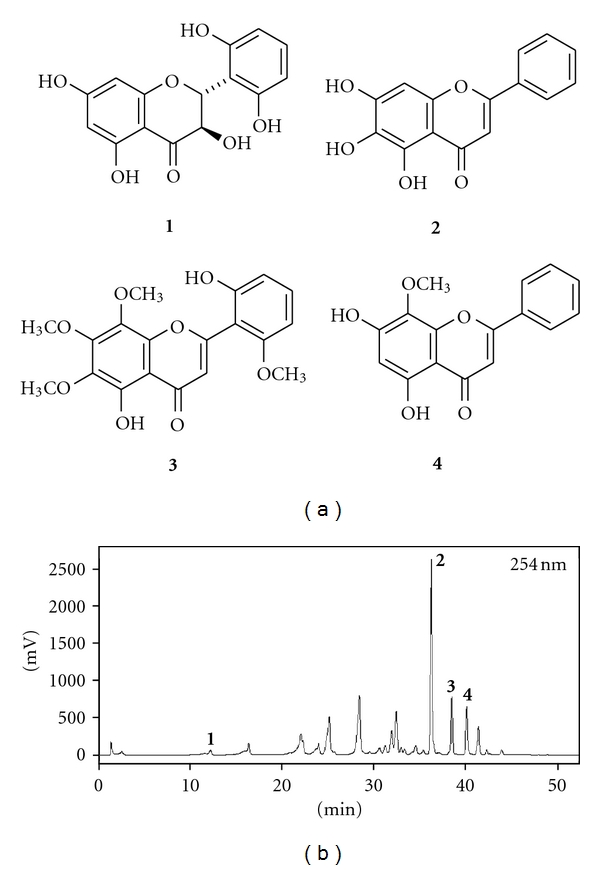
Chemical structures of compounds **1**–**4 **(a) and an HPLC chromatographic profile of the extract of *S. baicalensis *(b). The identity of the compounds was confirmed by coelution with authentic samples.

The structures of compounds **1**–**4** were identified as (2R, 3R)-2′,3,5,6′,7-pentahydroxyflavanone [19], baicalein [20], neobaicalein [21], and wogonin [20], respectively, by analysis of 1D- and 2D-NMR data and comparisons with reported molecules (Figure 1(a)). The presence and identification of (2R, 3R)-2′,3,5,6′,7-pentahydroxyflavanone (**1**), baicalein (**2**), neobaicalein (**3**), and wogonin (**4**) in the extract of *S. baicalensis* was confirmed by cochromatography with their respective isolated compounds on HPLC (Figure 1(b)).

### 3.2. Effects of ESB Extracts on the Cavernous Smooth Muscle Tone of Rabbit

**Figure 2:**
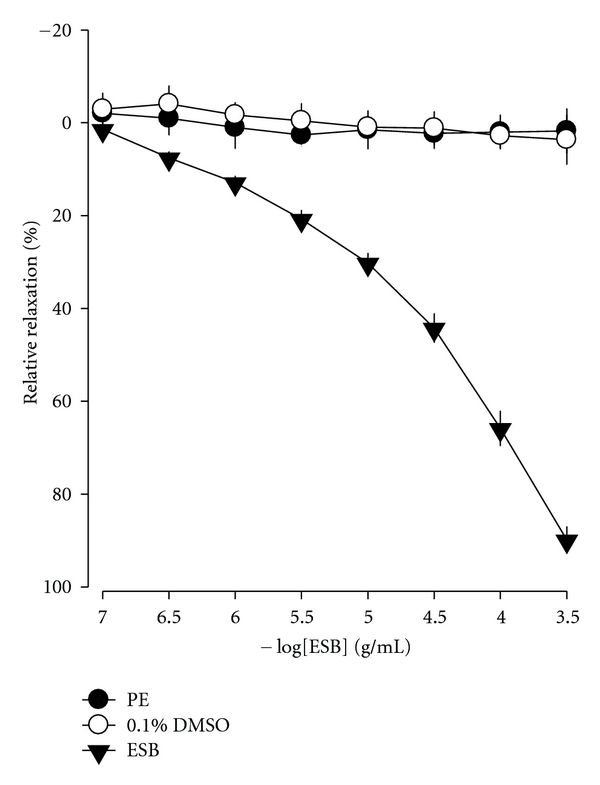
Effects of ESB on PE-precontracted endothelium-intact cavernosal smooth muscle strips. The vehicle treatment was 0.1% DMSO. Mean values ± S.E. (*n* = 7) are shown.

ESB relaxed the PE-precontracted endothelium-intact cavernous smooth muscle strip preparation in a dose-dependent manner (Figure 2). The maximal relaxant effect of ESB, achieved at a concentration of 0.3 mg/mL, was 89.85 ± 9.97% (Figure 2). DMSO (0.1%), a vehicle for ESB, had no significant relaxant effect.

### 3.3. Effect of NO Synthase Inhibition on ESB-Induced Cavernous Smooth Muscle Relaxation

**Figure 3:**
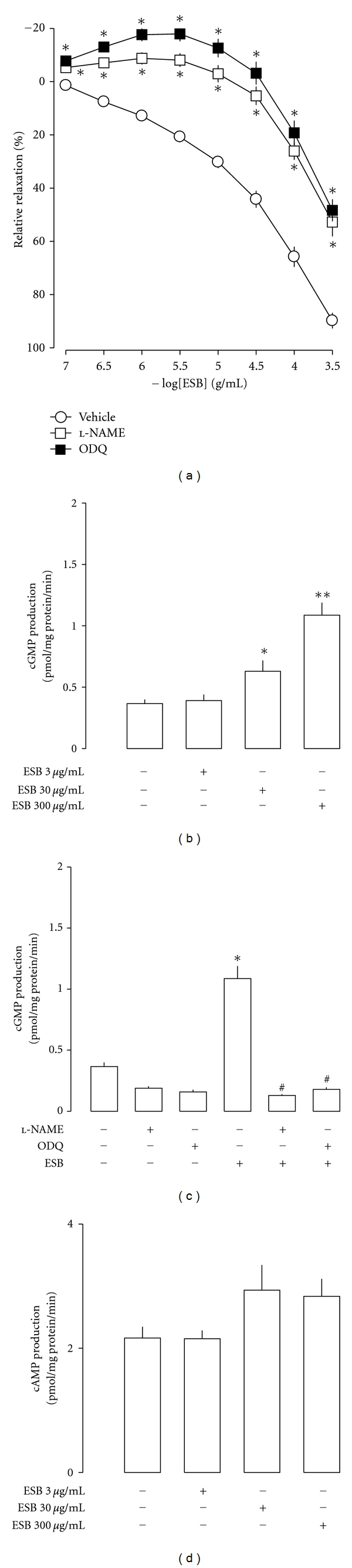
(a) Effects of 0.1 mM l-NAME and 10 *μ*M ODQ on ESB-induced relaxation of rabbit cavernosal smooth muscle strips. (c), (d) Effects of ESB on cGMP and cAMP level in crude homogenates of rabbit corpus cavernosum tissues. (b) Effects of l-NAME and ODQ on the ESB-induced increase of cGMP levels in crude homogenates of rabbit corpus cavernosum tissues. Mean values ± S.E. (*n* = 5) are shown. *P < 0.01, **P < 0.001 versus vehicle, ^#^ *P* < 0.001 versus ESB.

The effect of NO on ESB-induced cavernous smooth muscle relaxation was tested using l-NAME, an inhibitor of NO synthase. The addition of 0.1 mM l-NAME to the cavernous smooth muscle preparation significantly attenuated the ESB-induced relaxation effect (Figure 3(a)). This finding indicates that ESB elicits cavernous smooth muscle relaxation via NO-dependent signalling.

### 3.4. Effect of Inhibition of Guanylyl Cyclase Activity on ESB-Induced Cavernous Smooth Muscle Relaxation

To further verify the involvement of cGMP in EBS-induced cavernous smooth muscle relaxation, we tested the effect of ODQ, a soluble guanylyl cyclase (sGC) inhibitor. The addition of 10 *μ*M ODQ significantly attenuated the ESB-induced cavernous smooth muscle relaxation (Figure 3(a)), indicating that ESB induces cavernous smooth muscle relaxation via sGC-cGMP signalling. 

### 3.5. Effect of ESB on the Levels of cGMP and cAMP in Crude Tissue Homogenates of Rabbit Corpus Cavernosum

Incubation of corpus cavernosum strips with ESB increased the levels of cGMP in a concentration-dependent manner (Figure 3(b)). The addition of l-NAME and ODQ to the corpus cavernosum strip preparation blocked the ESB-induced increase of cGMP levels (Figure 3(c)). On the other hand, ESB had no effect on the cAMP levels of the corpus cavernosum smooth muscle (Figure 3(d)), indicating that ESB induces cavernous smooth muscle relaxation via NO-sGC-cGMP signalling.

### 3.6. Effects of K^+^ Channel Inhibitors on ESB-Induced Cavernous Smooth Muscle Relaxation

**Figure 4:**
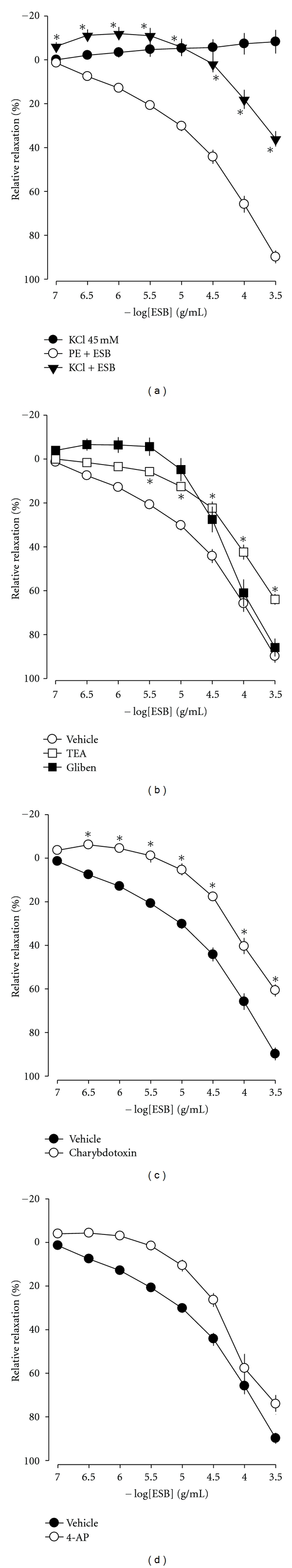
(a) Comparison of ESB-induced cavernous smooth muscle relaxation in high K-contracted and PE-precontracted rabbit cavernosum smooth muscle strips. The mean value ± S.E. are shown. KCl 45 mM (*n* = 5), KCl + ESB (*n* = 5), PE + ESB (*n* = 7). *P < 0.001 versus PE + ESB. (b) Effects of K^+^ channel blockade on ESB-induced rabbit cavernous smooth muscle relaxation. TEA, tetraethylammonium; gliben, glibenclamide. (c) Effect of K^+^-selective inhibitor on ESB-induced rabbit cavernous smooth muscle relaxation. Mean values ± S.E. (*n* = 5) is shown. *P < 0.001 versus vehicle. (d) Effect of 4-aminopyridine (4-AP) on ESB-induced rabbit cavernous smooth muscle relaxation. Mean values ± S.E. (*n* = 4) are shown.

ESB caused a concentration-dependent relaxation of PE-precontracted cavernous smooth muscle. However, the relaxant effect of ESB was greatly reduced in the presence of high K^+^ (45 mM) comparing with the presence of PE (*P* < 0.001, Figure 4(a)). To verify the involvement of K^+^ channels on ESB-induced cavernous smooth muscle relaxation, we treated a endothelium-intact cavernous smooth muscle strip with 1 mM TEA, a nonselective inhibitor of K^+^ channel blocker; 10 *μ*M glibenclamide, an adenosine triphosphate (ATP)-sensitive K^+^ (K_ATP_) channel blocker; 0.01 *μ*M charybdotoxin, a Ca^2+^-dependent K^+^ channel blocker; 100 *μ*M 4-AP, a voltage-dependent K^+^ channel blocker. ESB-induced cavernous smooth muscle strip relaxation was significantly attenuated by TEA and charybdotoxin, but not by 4-AP or glibenclamide (Figures 4(b), 4(c), and 4(d)). These findings suggest that ESB elicits cavernous smooth muscle relaxation via K^+^ channels, particularly the K_Ca_ channels.

### 3.7. Effects of Ca^2+^ on ESB-Induced Cavernous Smooth Muscle Relaxation

**Figure 5:**
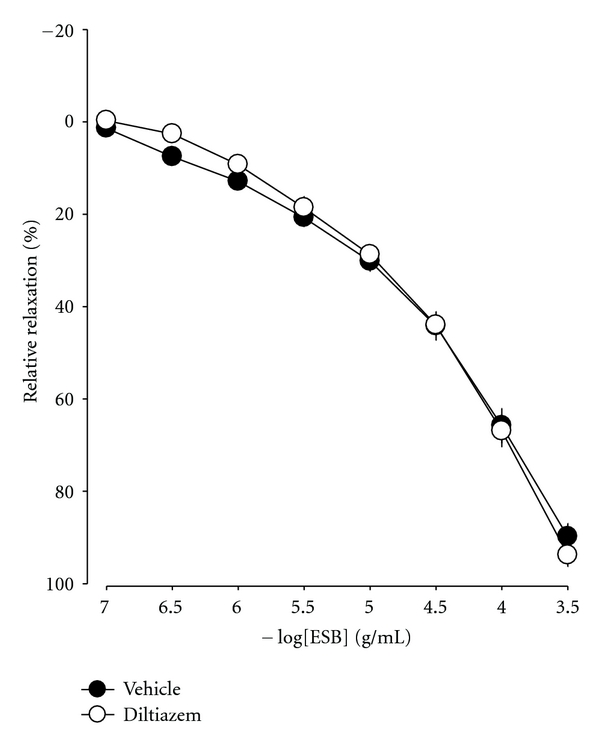
Effect of 10 *μ*M diltiazem on ESB-induced relaxation in PE-precontracted cavernosal smooth muscle strips. Mean values ± S.E. (*n* = 4) are shown.

To verify the involvement of Ca^2+^ channels on ESB-induced cavernous smooth muscle strip relaxation, we treated a smooth muscle strip with 10 *μ*M diltiazem, a voltage-gated Ca^2+^ channel blocker. Diltiazem had no effect on ESB-induced cavernous smooth muscle relaxation (Figure 5).

### 3.8. Effects of Prostacyclin on ESB-Induced Cavernous Smooth Muscle Relaxation

**Figure 6:**
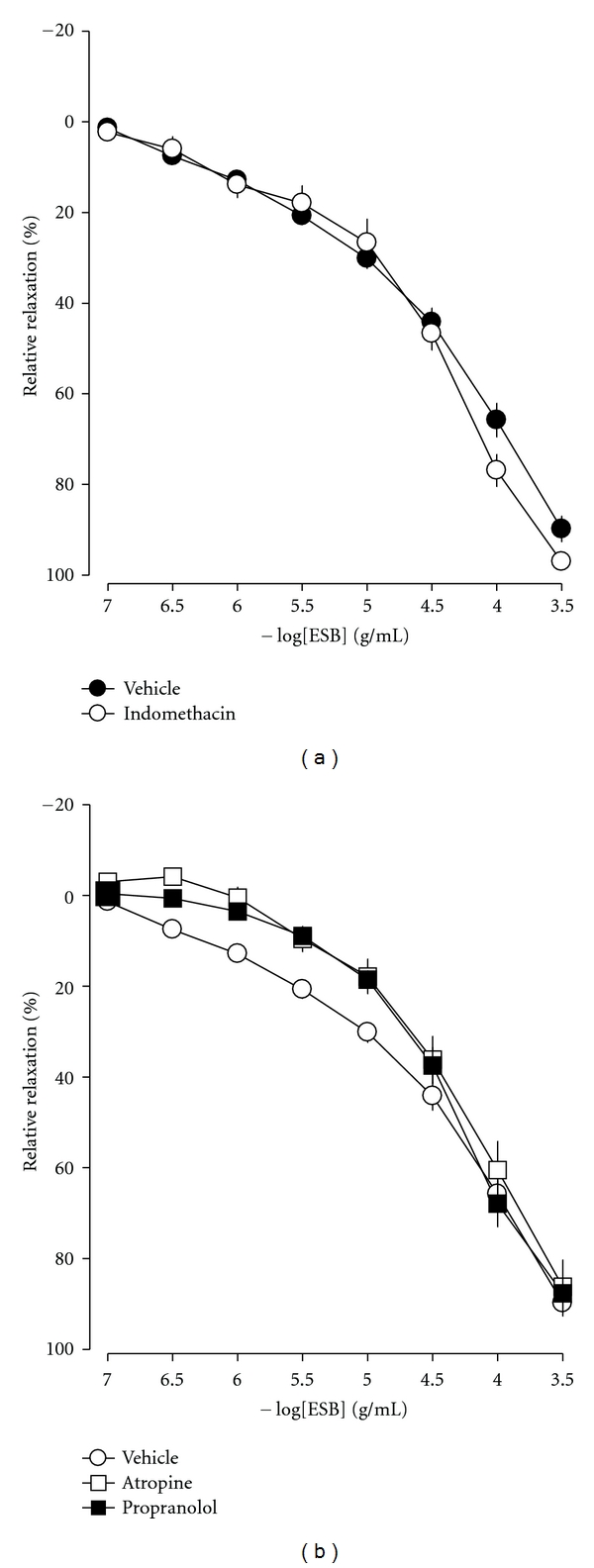
Effect of 10 *μ*M indomethacin, 1 *μ*M atropine, and 1 *μ*M propranolol on ESB-induced relaxation in PE-precontracted rabbit cavernosal smooth muscle strips. Mean values ± S.E. (*n* = 4) are shown.

To verify the involvement of prostacyclin (PGI_2_) on ESB-induced cavernous smooth muscle strip relaxation, we tested the effect of indomethacin, a nonspecific cyclooxygenase inhibitor. The relaxant effect of ESB on cavernous smooth muscle was not significantly influenced by pretreatment with 10 *μ*M indomethacin (Figure 6(a)). This finding suggested that cyclooxygenase products were not involved in the ESB-induced relaxation of cavernous smooth muscle.

### 3.9. Effects of Inhibitors of Adrenergic and Muscarinic Receptors on ESB-Induced Cavernous Smooth Muscle Relaxation

To verify the involvement of adrenergic and muscarinic receptors in ESB-induced cavernous smooth muscle strip relaxation, we tested the effects of atropine, an inhibitor of muscarinic receptors, or propranolol, a nonselective blocker of *β*-adrenoreceptors, on a smooth muscle strip. Pretreatment of the smooth muscle strip with 1 *μ*M atropine or 1 *μ*M propranolol had no effect on ESB-induced cavernous smooth muscle relaxation (Figure 6(b)). These findings indicate that adrenergic and muscarinic receptors are not involved in ESB-induced cavernous smooth muscle relaxation.

### 3.10. Effects of ESB on Muscle Tension in Perfused Rabbit Penile Tissue

**Figure 7:**
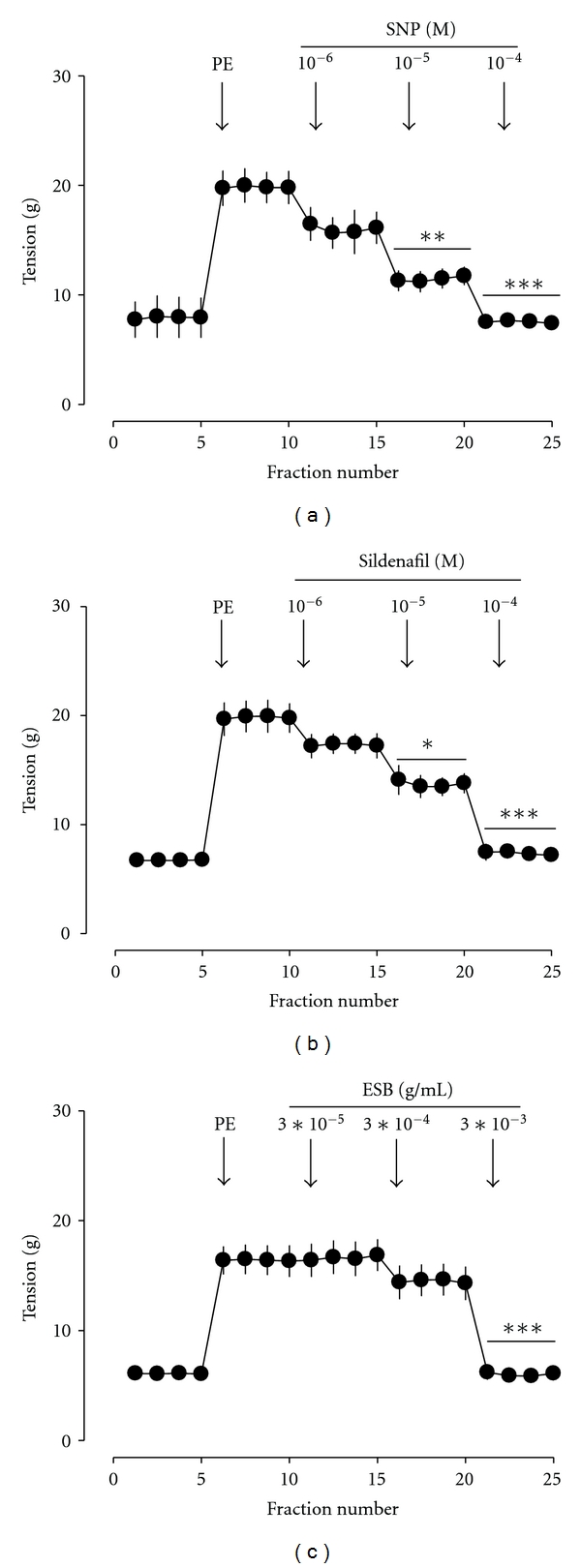
(a) Effect of SNP (a), sildenafil (b), and ESB (c) on corpus cavernosum smooth muscle tension in the perfused rabbit penile tissue. Each value shows mean ± S.E. (*n* = 6). *P < 0.05, **P < 0.01, ***P < 0.001 versus PE.

Penile corpus cavernosum smooth muscle tension was measured to confirm the relaxant effect of ESB on cavernous smooth muscle in the penile perfusion model. As shown in Figure 7, sodium nitroprusside (SNP, a NO donor) and sildenafil (type-5 PDE inhibitor) decreased the penile tissue tension in penile corpus cavernosum smooth muscle with the penile perfusion model in a dose-dependent manner, respectively. ESB also relaxed penile corpus cavernosum smooth muscle in a dose-dependent manner (Figure 7(c)).

## 4. Discussion

Nitric oxide, derived from vascular endothelial and neural sources, plays an essential role in the early steps of the normal cascade of relaxation of the penile vasculature and cavernous smooth muscle, and its action is mediated through the cGMP system [22, 23]. The present study shows that ESB elicits the relaxation of PE-precontracted cavernous smooth muscle strips in penile corpus cavernosum smooth muscle with penile strip chamber model as well as in penile perfusion model. The effects of various inhibitors were examined to verify the mechanism of ESB-induced relaxation. Pretreatment of cavernous smooth muscle with l-NAME, an inhibitor of NO synthase, and ODQ, an inhibitor of sGC, attenuated ESB-induced cavernous smooth muscle relaxation. In addition, these inhibitors blocked the ESB-induced increase of cGMP levels in the rabbit corpus cavernosum. These findings suggest that ESB-induced relaxation is closely associated with the activation of NO/cGMP signalling.

K^+^ channels also regulate corpus cavernosum smooth muscle tone [24]. K^+^ channel opener reduces the tissue tension or contractile force in response to the relaxation of the cavernous smooth muscle [25]. Vasodilators depend on the K^+^ channel mechanism, reducing their vasorelaxant effects in response to solutions containing high concentrations of K^+^, because an increase in extracellular K^+^ attenuates the K^+^ gradient across the plasma membrane, thereby rendering the K^+^-channel-activating mechanism ineffective [26]. Therefore, an impairment in K^+^ channel activity may contribute to erectile dysfunction, and, hence, we further investigated the possibility of K^+^ channel activation by ESB. The importance of K^+^-channel-mediated hyperpolarization in response to ESB was demonstrated by the potent difference of ESB on KCl-induced contraction compared to PE-induced contractions (*P* < 0.001). The ability of ESB to induce relaxation was greatly reduced in the presence of high K^+^ (45 mM). The relaxant effects of ESB, which were significantly reduced by TEA (K^+^ channel blocker) and charybdotoxin (Ca^2+^-dependent K^+^ channel blocker) [27], further suggest that the relaxant effects of ESB might be partly associated with K^+^ channel activities. However, ESB-induced cavernous smooth muscle relaxation was not blocked by the addition of glibenclamide (K_ATP_ channel blocker) or 4-AP (voltage-dependent K^+^ channel blocker) [28].

Calcium channels play an essential role in NO synthesis and release in endothelial cells. NO is known to activate K^+^ channels, either through the activity of cGMP-dependent protein kinase [29] or by direct opening of Ca^2+^-activated K^+^ channels without the requirement for cGMP [30]. cGMP is thought to cause the relaxation of smooth muscle by lowering the intracellular Ca^2+^ concentration, either by stimulating Ca^2+^-ATPase activity or through opening K^+^ channels, leading to hyperpolarization and subsequent reduction of Ca^2+^ influx through voltage-operated Ca^2+^ channels [29, 31]. This study showed that ESB-induced relaxation was not inhibited by diltiazem (voltage-gated calcium channel blocker) in cavernous smooth muscle.

Prostacyclin generated by endothelial cells has vasodilatory and antiaggregating effects on platelets [32]. To determine whether prostacyclin is involved in ESB-induced cavernous smooth muscle relaxation, we assayed the effect of indomethacin, a nonselective cyclooxygenase inhibitor, on ESB-induced relaxation. Our results show that ESB-induced cavernous smooth muscle relaxation was not inhibited by indomethacin, a prostacyclin synthesis blocker. To assess whether enhanced NO release by ESB was associated with the activation of muscarinic or adrenergic receptors, we examined the effects of atropine or propranolol on ESB-induced relaxation. The physiologic regulation of penile tumescence involves a balance between relaxation and contractile events. Relaxation is mainly promoted by endothelium-dependent mechanisms and stimulation of nitrergic nerves. In contrast, adrenergic neurotransmission has been reported as a promoter of penile flaccidity through the activation of *α*-adrenergic receptors [33]. Preincubation of the cavernous smooth muscle strips with atropine or propranolol did not affect ESB-induced relaxation. These findings indicate that ESB does not interact with muscarinic or *β*-adrenergic receptors.

Taken together, our findings suggest that the major mechanism underlying the relaxation of penile tissue by ESB involves the NO/cGMP system and Ca^2+^-sensitive K^+^ channels in the corpus cavernosum.

##  Conflict of Interests

The authors declared that there is no conflict of interests.
